# The Effects of Dietary *Spirulina platensisis* on Physiological Responses of Broiler Chickens Exposed to Endotoxin Stress

**DOI:** 10.3390/ani13030363

**Published:** 2023-01-20

**Authors:** Abdulaziz A. Alaqil, Ahmed O. Abbas

**Affiliations:** 1 Department of Animal and Fish Production, College of Agricultural and Food Sciences, King Faisal University, Al-Ahsa 31982, Saudi Arabia; 2 Department of Animal Production, Faculty of Agriculture, Cairo University, 7 Gamma St., Giza 12613, Egypt

**Keywords:** broilers, *Escherichia coli*, growth performance, antioxidant biomarkers, immunological parameters, cecal microflora, *Spirulina platensis*

## Abstract

**Simple Summary:**

Colibacillosis disease in poultry is induced by pathogenic *Escherichia coli* (EC) and causes serious economic drops in poultry industry. At the same time, using antibiotic therapies has been increasingly banned worldwide considering its compromising effects on human and animal health. This study investigates the prospective impacts of dietary *Spirulina platensisis* (SP) microalgae on the growth performance, antioxidant defense system, immune response, and intestinal microbiota in broiler chickens challenged with EC infection. Results showed that adding SP into broiler diets could overcome the deterioration effects of EC on broiler performance. The results may be innovative and applicable to reduce the cost of poultry production and improve the quality of poultry industry.

**Abstract:**

This study was proposed to highlight the impact of dietary *Spirulina platensis* (SP) supplementation in alleviating the deterioration effect of *Escherichia coli* (EC) on the growth performance, redox biomarkers, immune reaction, and hindgut microbial counts and acidosis in broiler chickens. Four hundred Cobb500, one-day-old, broiler chickens were deposited in battery cages (10 chicks per cage). The chicks were distributed into totally randomized 2 × 2 factorial treatments (10 replicate cages per treatment) from the day 22 to the day 42 of age. Birds of two of the groups were fed on a basal diet without SP supplementation (-SP groups), while birds of the other two groups were fed on a basal diet supplemented with 10 g/kg SP (+SP groups). At day 36th of age, birds in one of the -SP and +SP groups were challenged by an intraperitoneal (i.p.) injection with 10^7^ CFU/bird EC (O157:H7 strain) in 0.5 mL sterilized saline (+EC groups), whereas the other non-challenged groups were i.p. injected with 0.5 mL saline only (-EC groups). The current study results indicated that the boilers challenged with EC had a significant (*p* < 0.05) lower performance, poor antioxidant activity, immunosuppression, and higher numbers of pathogenic bacteria in the intestine when compared with the non-challenged birds. Dietary SP inclusion enhanced (*p* < 0.05) broiler growth, antioxidant activity, immune response, and intestinal beneficial bacteria and acidosis. Moreover, SP alleviated the reduction in all these parameters after exposure to EC infection. Therefore, diets containing 10 g/kg SP could be used as a promising approach to maximize broilers’ production and support their health, particularly when challenged with EC infection.

## 1. Introduction

Poultry meats have been considered as an essential source of animal protein in human food. Due to the massive production system, broiler chickens are virtually exposed to different types of stress factors such as cages, density, temperature, pests, parasites, and disease agents [1]. Such stressors may cause colibacillosis diseases induced by the activation of *Escherichia coli* (EC) in the digestive system of broiler chickens [2]. EC infection is one of the most critical challenges that counteracts the broiler growth and leads to a considerable economical drop for the business sector of meat production [3,4]. EC can invade the host via digestive or pulmonary tracts, then translocate across epithelium membranes to the bloodstream, and then colonize in various tissues [5].

Poultry infection with EC is commonly associated with several diagnostic symptoms such as airsacculitis, salpingitis, perihepatitis, peritonitis, pericarditis, and septicemia, which subsequently results in sudden death [4]. When EC is colonized in the intestinal tract, it disrupts the epithelial cells and adversely affects the absorption of nutrients [6]. It was reported that EC infection in broiler chickens increased the expression of some proinflammatory cytokines including interleukins (ILs), such as IL-1, IL-6, and IL8, to alert the immune system against the source of inflammation or infection [7]. In addition, EC caused oxidative stress and antioxidant-defense system depletion in the infected broiler chicks [8]. Reactive oxygen species (ROS) and free radicals were generated as a response to inflammation and immunological reaction in the EC-infected birds, and consequently, harm hematological and tissue cells [9]. Moreover, EC has a pathogenic component, lipopolysaccharide (LPS), which can induce immune stress in broilers [10]. It was reported that infection with avian pathogenic EC in broiler chickens had adverse effects on antibody production and cellular immunity [11] and caused an increase in the intestinal pathogenic bacteria against the beneficial bacteria [12].

*Spirulina platensisis* (SP) is a microscopic spiral-shaped blue-green alga existing in sea and river water [13]. Dried spirulina contains 50–70% protein with all essential amino acids, and 5–7% lipids including about 45% polyunsaturated fatty acids [14,15]. SP also comprises a wide range of fundamental nutrients, including vitamins (thiamine, riboflavin, nicotinamide, pyridoxine, folic acid, ascorbic acid, retinol, and α-tocopherol), minerals (potassium, calcium, chromium, copper, iron, magnesium, manganese, phosphorus, selenium, sodium, and zinc), and phytopigments (β-carotene, Xanthophylls, zeaxanthin, Chlorophyll, and phycocyanin) [13,16]. Additionally, SP has immunostimulant [17,18], anti-inflammation [19], anti-carcinogenic [20], and antioxidant activity [21] properties. It was reported that growth efficiency, nutrient digestibility, antioxidant enzyme activity, anti-inflammatory response, and the cecal microflora population of broiler chickens were remarkably enhanced by the dietary supplementation of the diets with SP at 0.25–1.0% [15,22,23]. Interestingly, SP supplementation into broiler diets was found to be beneficial under stress conditions. It was found that dietary supplementation with SP powder improved the growth, oxidation/reduction, and immunity of broiler chickens exposed to elevated temperature stress [24,25,26,27].

It is well-known that using antibiotic therapy in poultry breeding is increasingly not allowed worldwide due to the antibiotic resistance and residues, which compromise human and animal health [28]. This has prompted the scientists and poultry-business holders to try natural substitutes to overcome the problems related to antibiotics in feed. With the wide biological properties of SP, it could be used as a feed supplement to the broiler diets during the intensive production or stressful condition. According to our knowledge, there is a lack of sufficient studies discussing the possible effect of SP administration to broilers challenged by EC infection. Therefore, the current study aimed to highlight the prospective impact of feeding SP to broilers challenged by EC infection on their growth outputs, redox activity, immune response, and microbial count and acidosis of lower intestine.

## 2. Materials and Methods

### 2.1. Experimental Materials

A freeze-dried SP powder was obtained from a purveyor (Inner Mongolia Rejuve Biotech. Co., Ltd., Ordos, China) and kept at room temperature for the experiment. The analysis of the SP was conducted as the guidelines of the Association of Official Analytical Chemists (AOAC) [29]. The Folin–Ciocalteu assay was considered to determine the total polyphenols, using gallic acid equivalents (GAE) to determine the standard curve for the quantification of phenolic contents per g SP. In the same way, quercetin equivalents (QE) were used to analyze the total flavonoids per g SP [30]. In addition, the radical-scavenging 2,2-diphenyl-1-picrylhydrazyl (DPPH) test determined the total antioxidant activity according to the protocol described by Moukette Moukette et al. [31]. The chemical composition of SP is presented in Table 1.

*Escherichia coli* strain (O157:H7) was obtained from the Microbiological Resources Center (Cairo, Egypt) and propagated according to methods described in a previous work [32]. Briefly, colonies of the *E. coli* were cultivated at 37 °C in a MacConkey broth (Oxoid, Thermo Fisher Scientific Inc., Hampshire, UK) for 24 h. *E. coli* cells were harvested by centrifugation and then resuspended in a sterile saline solution to obtain a final colony forming unit (CFU) concentration of 2 × 10^7^ viable cells/mL.

### 2.2. Birds Management and Experimental Design

Four hundred Cobb500™ broiler chicks, males aged 1 day and weighing 48 ± 3.4 g (Al Watania Poultry Co., Riyadh, Saudi Arabia) were randomly placed in battery cages (10 chicks per cage) which distributed equally in two identical rooms (20 cages per room). These cages measured 125 × 90 × 60 cm with 1.5 mm thick wire floor. The chicks were kept under the same environmental standards of Cobb500 management guidelines [33] in both rooms. As per the Cobb500 guidelines, the feed rations were developed as mash using corn and soya meals. The broiler chicks were fed a starter diet for 1 week of age, a grower diet for 2–3 week of age, and a finisher diet for 4–6 week of age. Table 2 shows the diet ingredients and the nutritional composition determined by the AOAC methods [29]. Feed and water were unrestricted to the birds for the duration of the study.

From the 22nd to the 42nd day of age, the chicks were distributed into a totally randomized 2 × 2 factorial design according to the SP and EC treatments. Birds in each room were divided into two treatment groups (10 replicate cages per treatment group × 10 birds per cage); one of them was fed on a basal diet without SP supplementation (-SP groups), while the other one was fed on a basal diet supplemented with 10 g/kg SP (+SP groups). On the 36th day of age, all birds in the first room (200 birds belonged equally to the -SP and +SP groups) were challenged by an intraperitoneal (i.p.) injection with 10^7^ CFU/bird EC (O157:H7 strain) in 0.5 mL sterilized saline (+EC groups), whereas non-challenged birds in the second room (200 birds belonged equally to the -SP and +SP groups) were i.p. injected with 0.5 mL saline only (-EC groups) [34].

To ensure that the SP powder was consumed with the diet by birds, SP was mixed with 1 kg of the diet by hand, then this premix was thoroughly included in the remaining diet by a blender. The absence test of EC and salmonella infections was confirmed one day before the EC injection according to the previous work of da Rosa et al. [35]. After the EC challenge, frequent monitoring of birds was conducted to follow up on the progress of the pathogenic stress. If any symptoms such as low appetite, inactivity, mucous discharge, breathing disorder, etc., appeared with a fever (body temperature 43.5 °C), a humane endpoint was allowed by cervical dislocation of the suffering bird. The current study protocol was authorized by the research ethical committee of Saudi Arabia’s King Faisal University (Approval no. KFU-REC-2022-OCT-ETHICS275).

### 2.3. Growth Performance

The average body weight of broilers was determined at the 22nd day (BW_22_) and the 42nd day of age (BW_42_), and the body weight gain (BWG) was then calculated per replicate cage in each treatment group. Feed intake (FI) was determined by taking the leftover feed from the total amount introduced for each replicate in the treatment group. The feed conversion ratio (FCR) was then calculated for each replicate based on FI per unit of BWG.

### 2.4. Antioxidant Biomarkers

At the end of the trial (the 42nd day of age), blood samples were collected from the brachial vein of a bird in each replicate per treatment group (*n* = 10) and immediately put into tubes containing heparin. Plasma was separated by cooling centrifugation at 2000× *g* for 10 min and later used to analyze the antioxidant biomarkers. The glutathione reduced (GSH), the superoxide dismutase (SOD), and the malondialdehyde (MDA) reactions were assayed by colorimetric kits (Elabscience Biotechnology Inc., Houston, TX, USA), following a previous study [25]. The ceruloplasmin (CP) assay was performed using ELISA protocol [36] with specific kits for chicken (MyBioSource Inc., San Diego, CA, USA).

### 2.5. Immunological Parameters

At the end of the experiment on day 42 of age, blood samples were obtained from one bird per replicate in each treatment group (*n* = 10) and gently shaken with heparin into tubes. The heparinized sample was diluted with a brilliant cresyl blue (10:490 µL) and put on a Bright-Line™ hemocytometer slide (Ameri-can Optical, Buffalo, NY, USA). The slide was examined with a microscope at 200× magnification to distinguish and count the total white blood cells (TWBC) [37]. For measurement of heterophil-to-lymphocyte (H/L) ratio, a smear of the entire blood sample was stained by Hema-3 solutions (Fisher Scientific, Pittsburg, PA, USA) then approximately 200 leukocytes were differentiated under a microscope at 1000× magnification with oil immersion [38].

After that, the heparinized blood samples collected from the birds in each treatment group (*n* = 10) were used to measure the T and B lymphocyte proliferation (TLP and BLP) indexes, respectively [39]. Briefly, the peripheral blood mononuclear cells (PBMC) were layered on a separation medium then carefully harvested, washed twice, and resuspended in RPMI-1640 medium (Thermo Fisher Scientific, Waltham, MA, USA). The viable lymphocyte concentration was evaluated by using a Trypan Blue dye and re-adjusted at a final concentration of 10^6^ cells/mL in triplicates in microplate wells. The TLP and BLP were stimulated by Concanavalin-A mitogen and Lipopolysaccharide, respectively. After incubation with 3-(4,5-dimethylthiazol-2-yl)-2,5-diphenyltetrazolium (MTT), a solubilization solution (sodium dodecyl sulfates) was added to the cells. A microplate-ELISA reader was used to measure the absorbance for stimulated and non-stimulated samples at 570 nm (OD570) to compute the TLP and BLP indexes.

Furthermore, the anti-sheep red blood cells antibody (anti-SRBC Ab) titers were detected according to protocols outlined by Bhatti et al. [40] with a slight modification. In brief, a bird per replication in each group (*n* = 10) was injected at 35 day of age with 1 mL of washed-diluted SRBC (5% *v*/*v* in saline solution). Blood sera were collected at day 42 of age using centrifugation. Ten serial doubling dilutions of sera were prepared in a 96-well tray using saline solution. Each well was pipetted with 2% SRBC and then incubated at 37 °C for 30 min. The Ab titer was reported as log_2_ of the reciprocal values of the last dilution showing certain agglutination.

### 2.6. Intestinal Microbial Count and Acidosis

After the treatments (42 day of age), ceca samples were obtained from a bird per replicate in each treatment (*n* = 10) to analyze the microbial population, including lactic acid bacteria (LAB), *Salmonella* (SLM), and EC, as per methods described by Al-Khalaifah et al. [41]. In summary, broiler chickens were slaughtered, weighed, and washed with 1:2 diluted disinfectant under refrigerated conditions. The abdominal feather was removed, and the area was sterilized with 70% ethanol. The abdomen area was dissected using a sterile scissor and forceps to carefully harvest out the digestive system. The caecum was surgically removed from the lower intestine, and its weight was recorded. Caecum contents were extracted by splitting the caecum lengthwise, gently scraping the epithelium, and squeezing the contents into a sterile petri dish filled with 0.85% sterile saline. The crude caecum extract was homogenized in a sterile stomacher bag for 3 min. Serial dilutions of the extract were prepared, and 0.1 mL of the dilutions were flattened onto the surface of respective agar plates for each analysis. The agar plates contained de Man, Rogosa, Sharpe (MRS), Xylose-Lysine-Desoxycholate, or Brilliance *E. coli* selective media (Oxoid, Thermo Fisher Scientific Inc., Hampshire, UK) for LAB, SLM, or EC counts, respectively. The plates were incubated anaerobically for 48 h at 30 °C for LAB, while for both SLM and EC, the plates were incubated aerobically for 24 h at 37 °C. Finally, colony counts (CFU/mL) were detected and transformed into log values.

A colon acidosis test was conducted on a broiler randomly selected per replication in each treatment (*n* = 10) on the last day of the experiment (42 day of age). Colon digesta was placed into tubes, and the pH value was detected using a pH meter (Hanna Instruments, Inc., Smithfield, RI, USA).

### 2.7. Statistical Analysis

Data were arranged following a 2 × 2 factorial design, considering SP treatment (-SP versus +SP), EC challenge (-EC versus +EC), and their interaction (SP × EC) as the main factors in the analysis. Variables of growth performance, antioxidant biomarkers, immunological parameters, microbial counts, and caecal acidosis were analyzed by the two-way analysis of the general linear model (GLM) procedure using the IBM SPSS Statistics version 22 (IBM Corp., NY, USA) [42]. Means of the interaction with the pooled standard error of means (SEM) and *p*-values were displayed. Statistical significance was considered to exist at *p*-value < 0.05. When a statistical significance for SP, EC, or their interaction effect appeared, the differences between the groups were separated using “Duncan’s post hoc” test.

## 3. Results

### 3.1. Growth Performance

The effect of dietary SP supplementations on the growth performance of broilers challenged by EC infection is shown in Table 3. The results show significant (*p* < 0.05) effects for the SP treatment, EC infection, and their interaction on all parameters of broiler growth performance. The final BW at 42 day of age, BWG, and FI were significantly (*p* < 0.05) reduced by 20%, 28%, and 8%, respectively, while the FCR was significantly (*p* < 0.05) increased by 29% in the EC-infected broilers compared to the control group (-EC/-SP group). On the contrary, the SP treatment significantly (*p* < 0.05) improved the BW_42_, BWG, and FI, by approximately 5%, 8%, and 6%, respectively, when compared with the control group. Furthermore, the broilers supplemented with SP expressed a significant (*p* < 0.05) resistance to the reduction in their performance when challenged by EC infection.

### 3.2. Antioxidant Biomarkers

The effect of dietary SP supplementations on the antioxidant biomarkers of broilers challenged by EC infection is presented in Table 4. The results demonstrate that SP treatment significantly (*p* < 0.05) increased the GSH level and SOD activity, while it decreased the levels of CP and MDA in the plasma. When the broilers were challenged with EC, the GSH and SOD were significantly (*p* < 0.05) reduced by approximately 1.4- and 1.6-fold, respectively, compared to the control group. In contrast, the levels of CP and MDA were significantly (*p* < 0.05) increased by two-fold in the EC-infected broilers compared to non-EC-infected broilers. A significant interaction effect existed for SP × EC on some antioxidant biomarkers, indicated by modulating the negative impact of EC infection on GSH and CP levels in the SP-supplemented birds.

### 3.3. Immunological Parameters

The effect of dietary SP supplementations on the immune response of broilers challenged by EC infection is shown in Table 5. Results showed a significant increase in all immunological parameters due to the SP treatment, while a significant decrease occurred in all parameters due to the challenge with the EC infection (*p* < 0.05). A significant interaction effect was observed for SP × EC on all parameters except the TWBC. The EC infection significantly (*p* < 0.05) increased the H/L ratio by 2.4-fold compared to non-EC broilers without SP supplementation. In the SP-supplemented broilers, the elevation in the H/L ratio by infection was reduced again by 1.2-fold. In contrast, a reduction of 73%, 50%, and 44% was found in the TLP, BLP index, and the Ab-titers against SRBC in the EC-infected broilers compared to the non-infected broilers without SP supplementation. However, the SP supplementat1ion significantly (*p* < 0.05) ameliorated the adverse impact of EC infection on these immunological parameters in challenged birds.

### 3.4. Microbial Counts and Acidosis

The effect of dietary SP supplementations on the immune response of broilers challenged by EC infection is presented in Table 6. SP supplementation significantly (*p* < 0.05) increased intestinal acidosis and inhibited the growth of SLM and EC, while it increased the LAB population. On the contrary, broilers challenged with EC infection showed a significant increase in the intestinal pH, SLM, and EC, and a significant decrease in the LAB counts. There was a significant interaction effect for SP × EC on all these parameters except EC counts. When the broiler diets were supplemented with SP, the elevation in the intestinal pH and SLM counts were lowered, and the LAB growth was promoted again in the EC-infected birds.

## 4. Discussion

The present work was designed to assess the potential effect of SP inclusion into broiler diets on their growth, antioxidant activity, immune response, and intestinal microbial count and acidosis of broiler chickens challenged by EC infection. We used a selected level of SP (10 g/kg as fed) which was recommended based on a previous study [25]. As per the results of this study, dietary SP supplementation at levels of 5, 10, and 15 g/kg to broilers exposed to heat stress relieved the deterioration that occurred in the productive performance, carcass quality, redox status, and blood metabolites, with the best outputs recorded in the chickens fed 10 g/kg SP.

EC infection occurs in broiler chickens when they are exposed to substances or tools contaminated with an avian pathogenic strain of EC per se [2] or exposed to various stress factors that activate EC in their digestive system [1]. The current study manifested that EC infection deteriorates the growth performance of challenged broilers. The depression in feed intake reached 8% in EC-challenged broilers compared to non-EC-challenged broilers. The repression of feeding behavior may be due to the harmful action of nitric oxide and interleukin cytokines on the brain tissue of EC-infected birds [34,43]. The decrease in feed intake can directly cause a significant decrease in BWG and FCR [44]. We recorded a reduction of 20% and 28% in the BW_42_ and BWG, respectively, and an increase of 29% in the EC-infected broilers. Similarly, Boratto et al. [37] found a reduction in BWG by 16% and a reduction in FI by 7%, as well as an increase in FCR by 9% in the EC-infected broilers compared to non-infected birds [45].

The impairment in the broiler growth performance induced by EC infection in the present study was accompanied by an approximately 1.5-fold reduction in the GSH and SOD antioxidants, and a 2-fold increase in the CP and MDA levels. It was reported that *E. coli* produces excess free radicals and ROS that can induce irreversible damage in the antioxidant defense system and, in turn, dramatically reduce the productive outputs in poultry [46]. In addition, da Rosa et al. [8] reported that chickens infected with *E. coli* represent poor growth aspects and attributed this to the oxidative stress remarked by high levels of serum and hepatic ROS. In contrast, SP treatment improved the growth performance of both non-challenged and challenged birds with EC infection (Table 3). The SP treatment also increased the antioxidant activity and decreased the oxidative stress in the broilers (Table 4). Similar effects for SP were reported on the productive performance, redox activity, and immune response in normal broiler chickens [15,22,23] and in broilers exposed to heat stress [24,25,26,27]. The positive effects of the SP treatment may be accounted for by its antioxidant and anti-inflammatory effects [47]. The antioxidant properties of SP were evidenced via the chemical analysis of SP in our study (Table 1) and other studies [48]. The polyphenols and flavonoids that existed in the SP are sufficient to promote the scavenging action against the free radicals produced by EC in the infected broilers.

SP supplementation in poultry nutrition improved the performance not only by their biological functions but also by their rich nutrients [13,16]. According to the chemical analysis of SP in our study, it contained high amounts of protein (56.4%) and amino acids (Table 1). It calculatedly means that supplemental 1% (10 g/kg) SP can increase the crude protein of the diet by 0.5% (5.6 g/kg). Thus, the improved performance of broilers treated with SP could be attributed to the contribution of SP to increase the high-quality protein content of the diets.

The EC challenge in the broilers dramatically suppressed all immunological parameters examined in this study. One of the major reasons for disturbing the immune system in EC-challenged birds is the presence of lipopolysaccharides (LPS) on the outer membrane of EC [49]. LPS can produce large amounts of ROS and proinflammatory cytokines, which consequently disrupt the normal function of target cells, including immune cells [50]. After the challenge, the lymphocyte proliferation was poor and a reduction of 73% and 50% was observed in the TLP and BLP, respectively. These data are consistent with earlier studies that found a suppressive effect of EC on the proliferative responses of avian splenocytes [51,52]. On the contrary, SP is well recognized for its anti-inflammation and immunomodulation properties [23,48,53]. When the broilers were supplemented with SP, most of the immunological parameters were remarkably improved in both challenged and non-challenged broilers. The positive effect of SP on broiler immunity could be referred to its high nutritional value, especially with protein [54]. In addition, the presence of essential amino acids in SP, as indicated in Table 1, may contribute to several immunological functions such as the activation of T and B lymphocytes, the regulation of immune-cellular redox status, the lymphocyte proliferation, and the production of antibodies [55,56].

The intestinal microbial population and acidosis were also investigated in this study to illustrate the effectiveness of SP on the broilers’ health and their ability to resist pathogens before and after the EC challenge. Our results indicated that EC infection not only deteriorated the growth performance and suppressed the immune response, but also exhibited an expansion of harmful bacteria such as SLM and inhibited the beneficial bacteria such as LAB (Table 6). However, it is fortunate that SP supplementation contrasted with these negative effects in the EC-challenged broilers. Interestingly, a recent in vitro study pointed out that spirulina extracts have antimicrobial activity against various pathogenic microorganisms [57]. It was reported that SP bioactive compounds, such as phenols, act on the cell integrity and permeability of pathogens, thus restraining their invasion, attachment, motility, and biofilm formation into animal guts [58,59]. In addition, the development of LAB in the SP-supplemented broiler groups created an acidic-pH micro-ecology coming from the release of volatile fatty acids (VFAs) in the caeca and colon [41]. These lipophilic VFAs penetrate the out-membrane of the pathogenic bacteria and produce hydrogen ions, which in turn lead to eliminating or destroying the bacterial cell [60].

## 5. Conclusions

The present study results showed that supplementing diets with 10 g/kg SP in broiler chickens challenged with EC could overcome the deterioration in their performance, antioxidant defense, and immune response, and could promote healthy microbiota in their intestines. This study features the novelty of SP use in poultry feed rations as an alternative to antibiotics considering the problems related to antibiotic resistance and residuals in animal and human feed.

## Figures and Tables

**Table 1:** The results of *Spirulina platensisis* (SP) chemical analysis.

Item	Contents Per 100 g SP
Moisture (g)	5.6
Crude protein (g)	56.4
Total lipids (g)	7.2
Carbohydrate (g)	14.2
Crude fiber (g)	0.02
Total ash (g)	7.5
Energy (MJ)	43.6
Calcium (mg)	436.3
Phosphorus (mg)	124.5
Sodium (mg)	220.1
Potassium (mg)	167.8
Iron (mg)	11.5
Zinc (mg)	2.4
Total polyphenols (mg GAE/g) ^1^	22.1
Total flavonoids (mg QE/g) ^1^	6.7
Total antioxidant activity (%) ^2^	29.2
Essential amino acids ^3^	
Isoleucine	6.6 g
Leucine	8.3 g
Valine	6.6 g
Lysine	4.8 g
Tryptophan	1.1 g
Phenylalanine	4.7 g
Methionine	2.4 g
Threonine	5.3 g

^1^ GAE or QE are gallic acid or quercetin equivalent determined per g of the SP powder. ^2^ Determined as a percentage of radical scavenging activity of the SP. ^3^ According to the commercial supplier (not analyzed).

**Table 2:** Ingredients and nutritional composition of the starter (0–7 day), grower (8–21 day) and finisher (22–42 day) feed rations introduced to the Cobb500 broiler chickens.

Ingredients (g/kg as Fed)	Starter Diet	Grower Diet	Finisher Diet
Corn	607.0	654.0	693.0
Gluten meal	70.0	50.0	50.0
Soybean meal, 48% CP	289.0	243.0	203.0
Soybean oil	0.0	20.0	22.0
Di-calcium phosphate	4.0	4.0	4.0
Limestone	20.0	19.0	18.0
Salt	4.5	4.5	4.5
Vitamin–Mineral Premix ^1^	5.5	5.5	5.5
Calculated nutrients			
Metabolizable energy (MJ/kg)	12.6	13.1	13.3
Lysine (g/kg)	12.1	11.6	10.4
Methionine (g/kg)	4.8	4.7	4.3
Calcium (g/kg)	9.1	8.6	8.1
None phytase phosphorus (g/kg)	4.5	4.2	4.1
Determined nutrients			
Dry matter (g/kg)	906.0	901.0	908.9
Total ash (g/kg)	55.0	53.0	39.1
Crude protein (g/kg)	229.8	199.8	184.6
Crude fat (g/kg)	58.3	77.5	83.4
Crude fiber (g/kg)	32.0	35.0	35.8

^1^ Premix (contents per kg of the basal diet): 10 KIU vit A, 5 KIU vit D_3_, 65 IU vit E, 3 mg vit K, 3 mg vit B_1_, 9 mg vit B_2_, 4 mg vit B_6_, 0.02 mg vit B_12_, 0.20 mg biotin, 20 mg niacin, 15 mg pantothenic acid, 2 mg folic acid, 500 mg choline chloride; 100 mg Mn, 100 mg Zn, 40 mg Fe, 15 mg Cu, 1 mg Iodine, and 0.35 mg Se.

**Table 3:** Effect of dietary *Spirulina platensisis* (SP) supplementation on the growth performance of broilers challenged by *Escherichia coli* (EC) infection.

Parameters	-SP		+SP		SEM	*p*-Value		
	-EC	+EC	-EC	+EC		SP	EC	SP × EC
BW_22_ (g)	632.7	642.0	621.5	640.3	14.45	0.658	0.337	0.744
BW_42_ (g)	2380.9 ^b^	1898.7 ^c^	2508.7 ^a^	2372.5 ^b^	17.79	<0.001	<0.001	<0.001
BWG (g)	1748.2 ^b^	1256.7 ^c^	1887.2 ^a^	1732.1 ^b^	20.60	<0.001	<0.001	<0.001
FI (g)	150.3 ^b^	138.0 ^c^	159.4 ^a^	162.5 ^a^	1.26	<0.001	0.001	<0.001
FCR	1.80 ^c^	2.32 ^a^	1.77 ^c^	1.97 ^b^	0.031	<0.001	<0.001	<0.001

Treatment groups: -SP, a broiler group that received a basal diet without SP supplementation; +SP, a broiler group that received a basal diet supplemented with 10 g/kg SP; -EC, a broiler group that was not challenged by EC infection; +EC, a broiler group that was challenged by EC infection. Parameters: BW_22_, the body weight of broilers on the 22nd day of age; BW_42_, the body weight of broilers at the 42nd day of age; BWG, body weight gain; FI, feed intake; FCR, feed conversion ratio. Data are expressed as means of 10 replicate cages per treatment group (*n* = 10) with the pooled standard error of means (SEM), and the probability (*p*-value) of SP, EC, and their interaction (SP × EC) effects. Means with uncommon superscripts (a, b, etc.) within a parameter significantly differ at *p* ≤ 0.05.

**Table 4:** Effect of dietary *Spirulina platensisis* (SP) supplementation on the antioxidant biomarkers of broilers challenged by *Escherichia coli* (EC) infection.

Parameters	-SP		+SP		SEM	*p*-Value		
	-EC	+EC	-EC	+EC		SP	EC	SP × EC
GSH (nM/mL)	27.63 ^b^	20.32 ^c^	37.47 ^a^	26.04 ^b^	0.604	<0.001	<0.001	0.002
SOD (U/mL)	6.13 ^b^	3.33 ^c^	9.53 ^a^	6.24 ^b^	0.212	<0.001	<0.001	0.249
CP (pg/mL)	1.05 ^c^	2.17 ^a^	0.92 ^d^	1.70 ^b^	0.022	<0.001	<0.001	<0.001
MDA (nM/mL)	2.54 ^c^	5.33 ^a^	1.47 ^d^	3.44 ^b^	0.210	<0.001	<0.001	0.056

Treatment groups: -SP, a broiler group that received a basal diet without SP supplementation; +SP, a broiler group that received a basal diet supplemented with 10 g/kg SP; -EC, a broiler group that was not challenged by EC infection; +EC, a broiler group that was challenged by EC infection. Parameters: GSH, glutathione reduced; SOD, superoxide dismutase; CP, ceruloplasmin; MDA, malondialdehyde. Data are expressed as means of 10 birds per treatment group (*n* = 10) with the pooled standard error of means (SEM), and the probability (*p*-value) of SP, EC, and their interaction (SP × EC) effects. Means with uncommon superscripts (a, b, etc.) within a parameter significantly differ at *p* ≤ 0.05.

**Table 5:** Effect of dietary *Spirulina platensisis* (SP) supplementation on the immunological parameters of broilers challenged by *Escherichia coli* (EC) infection.

Parameters	-SP		+SP		SEM	*p*-Value		
	-EC	+EC	-EC	+EC		SP	EC	SP × EC
TWBC (K/µL)	55.73 ^b^	35.37 ^d^	62.66 ^a^	43.85 ^c^	1.828	<0.001	<0.001	0.675
H/L ratio	0.33 ^c^	0.78 ^a^	0.33 ^c^	0.66 ^b^	0.021	0.007	<0.001	0.010
TLP index	3.71 ^b^	1.02 ^c^	5.19 ^a^	3.54 ^b^	0.169	<0.001	<0.001	0.004
BLP index	2.48 ^b^	1.23 ^c^	2.90 ^a^	2.70 ^ab^	0.082	<0.001	<0.001	<0.001
Ab titer (log_2_)	8.21 ^b^	4.61 ^d^	9.01 ^a^	6.72 ^c^	0.270	<0.001	<0.001	0.020

Treatment groups: -SP, a broiler group that received a basal diet without SP supplementation; +SP, a broiler group that received a basal diet supplemented with 10 g/kg SP; -EC, a broiler group that was not challenged by EC infection; +EC, a broiler group that was challenged by EC infection. Parameters: TWBC, total white blood cells (K = 10^3^); H/L ratio, heterophils to lymphocytes ratio; TLP index, T-lymphocytes proliferation index; BLP index, B-lymphocytes proliferation index; Ab titer, antibody titer against sheep red blood cells. Data are expressed as means of 10 birds per treatment group (*n* = 10) with the pooled standard error of means (SEM), and the probability (*p*-value) of SP, EC, and their interaction (SP × EC) effects. Means with uncommon superscripts (a, b, etc.) within a parameter significantly differ at *p* ≤ 0.05.

**Table 6:** Effect of dietary *Spirulina platensisis* (SP) supplementation on the microbial counts and colon acidosis of broilers challenged by *Escherichia coli* (EC) infection.

Parameters	-SP		+SP		SEM	*p*-Value		
	-EC	+EC	-EC	+EC		SP	EC	SP × EC
Intestinal pH	7.14 ^b^	7.66 ^a^	5.83 ^d^	6.89 ^c^	0.060	<0.001	<0.001	<0.001
LAB (CFU/g) *	2.98 ^c^	2.56 ^d^	4.47 ^a^	3.03 ^b^	0.001	<0.001	<0.001	<0.001
SLM (CFU/g) *	1.11 ^c^	2.89 ^a^	0.03 ^d^	1.35 ^b^	0.023	<0.001	<0.001	<0.001
EC (CFU/g) *	7.59 ^b^	8.20 ^a^	6.81 ^c^	7.38 ^b^	0.117	<0.001	<0.001	0.861

Treatment groups: -SP, a broiler group that received a basal diet without SP supplementation; +SP, a broiler group that received a basal diet supplemented with 10 g/kg SP; -EC, a broiler group that was not challenged by EC infection; + EC, a broiler group that was challenged by EC infection. Parameters: LAB, lactic acid bacteria; SLM, *Salmonella*; EC, *Escherichia coli*. * Log values of microbial colony forming unit (CFU) concentration. Data are expressed as means of 10 birds per treatment group (*n* = 10) with the pooled standard error of means (SEM), and the probability (*p*-value) of SP, EC, and their interaction (SP × EC) effects. Means with uncommon superscripts (a, b, etc.) within a parameter significantly differ at *p* ≤ 0.05.

## Data Availability

The data presented in this study are available on request from the corresponding author.
